# Deep learning based load station inspection for smart manufacturing with limited data

**DOI:** 10.1007/s00170-026-18606-4

**Published:** 2026-07-01

**Authors:** Yasir Ijaz, Sonya Coleman, Dermot Kerr, Nazmul Siddique, Cormac McAteer, Bryan Baker, Khoi Nguyen

**Affiliations:** 1 School of Computing, Engineering and Intelligent Systems, Ulster University, Northland Road, Londonderry, BT48 7JL UK; 2 Seagate Technology, Springtown Industrial Estate, Londonderry, BT48 0LY UK

**Keywords:** Industrial Load station inspection, Smart manufacturing, Transfer learning, Low-resource data, Computer vision

## Abstract

Fast and accurate industrial Load Station (LS) inspection is crucial in manufacturing environments. This study addresses the challenges of deploying a deep learning-based solution for LS inspection in semiconductor wafer handling, where the Load Station must be properly aligned and occlusion-free before pin pack assembly operations. A significant challenge in industrial settings is data scarcity, as collecting and annotating large amounts of datasets is often impractical due to operational constraints and the rarity of abnormal conditions. This study examines how training data size affects inspection reliability in a real-world smart manufacturing context. The experiments utilise YOLOv5 and YOLOv8 variants across five training set sizes to determine minimum data requirements for reliable deployment, evaluated under 5-fold cross-validation with multiple random seeds. Our results demonstrate that YOLOv8 achieves superior data efficiency, with only 40 samples per class (*T*-40), YOLOv8n achieves *0.981 ± 0.014* inspection accuracy and *0.842 ± 0.050* mean Average Precision (mAP@0.5) on a held-out real test set, while YOLOv5 requires substantially more training data to achieve comparable performance. Smaller variants (YOLOv8n, YOLOv8s) consistently outperform larger models in this data-scarce environment. An ablation study confirms that combining real and synthetic obstruction samples is essential, and the approach is validated on an operational semiconductor manufacturing dataset, providing practical, statistically grounded recommendations for deploying deep learning inspection systems when training data are limited.

## Introduction

In the modern era of Industry 4.0, digitalisation and automation are core components of high-speed and intelligent manufacturing systems, ensuring reliable production. These systems demand advanced technologies, including artificial intelligence, machine learning and computer vision, to facilitate real-time decision-making for quality inspection [13].

The conversion from conventional manufacturing to smart factories improves product quality and productivity, enabling automated inspection as an essential element of contemporary production lines [21]. Studies indicate that automation within the industrial sector enhances operational efficiency by 20-30% and improves resource utilisation [22]. Visual inspection systems that utilise deep neural networks (DNNs) have demonstrated exceptional efficacy in object recognition and localisation for automated quality control [16]. However, a main challenge is the necessity for extensive training data to train DNNs. In contrast, while extensive datasets such as ImageNet or COCO are readily accessible for generic object detection in real life, manufacturing operations face significant limitations in data acquisition. The primary challenge is that rare abnormal conditions may arise, production cannot be suspended for data collection, and expert annotation is both time-consuming and costly [23, 24].

**Fig. 1:**
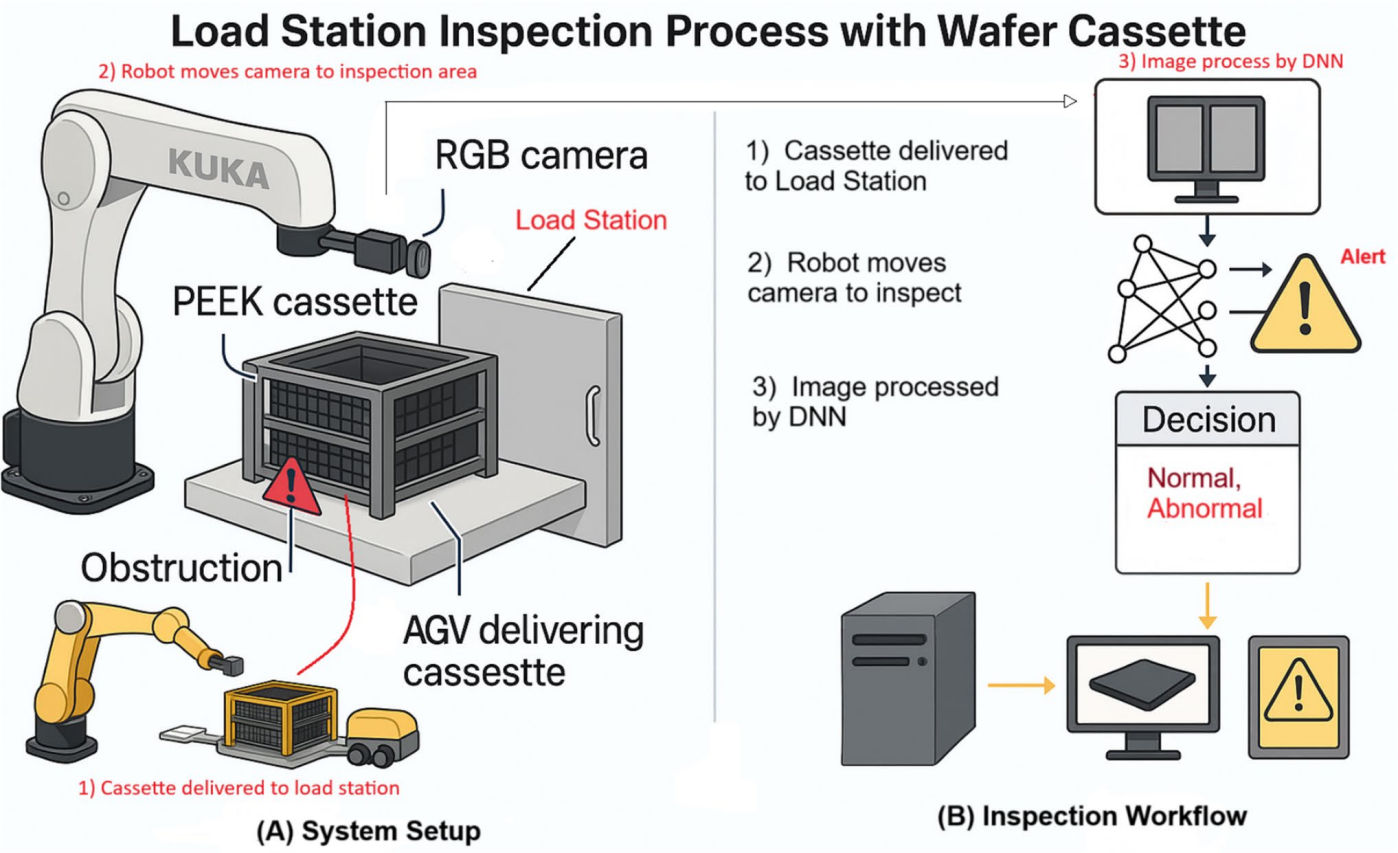
Schematic for Load Station inspection system: KUKA robot with mounted camera inspecting the High-Resolution Inspection Load Station prior to pin-pack assembly: (**A**) System Setup: KUKA robotic arm, RGB camera with controlled lighting, Load Station, PEEK cassette placement area. (**B**) Inspection workflow: the robot approaches the LS, captures an image, processes it through the trained DNN, and outputs the inspection decision (*Normal*/*Abnormal*)

In the manufacturing of semiconductor wafers, where accuracy is very important and mistakes are costly, this lack of data problem is especially critical. In High-Resolution Inspection (HRI) systems for wafer handling, the readiness of each inspection station must be confirmed prior to starting operation. Human operators conduct manual inspections, which is prone to errors, particularly during repetitive tasks over prolonged shifts. An automated vision-based inspection system provides consistency and reliability; the DNN-based system for Load Station inspection overview is illustrated in Fig. 1. However, its implementation typically depends on large amounts of data; we address this constraint by operating under limited training data.

**Fig. 2:**
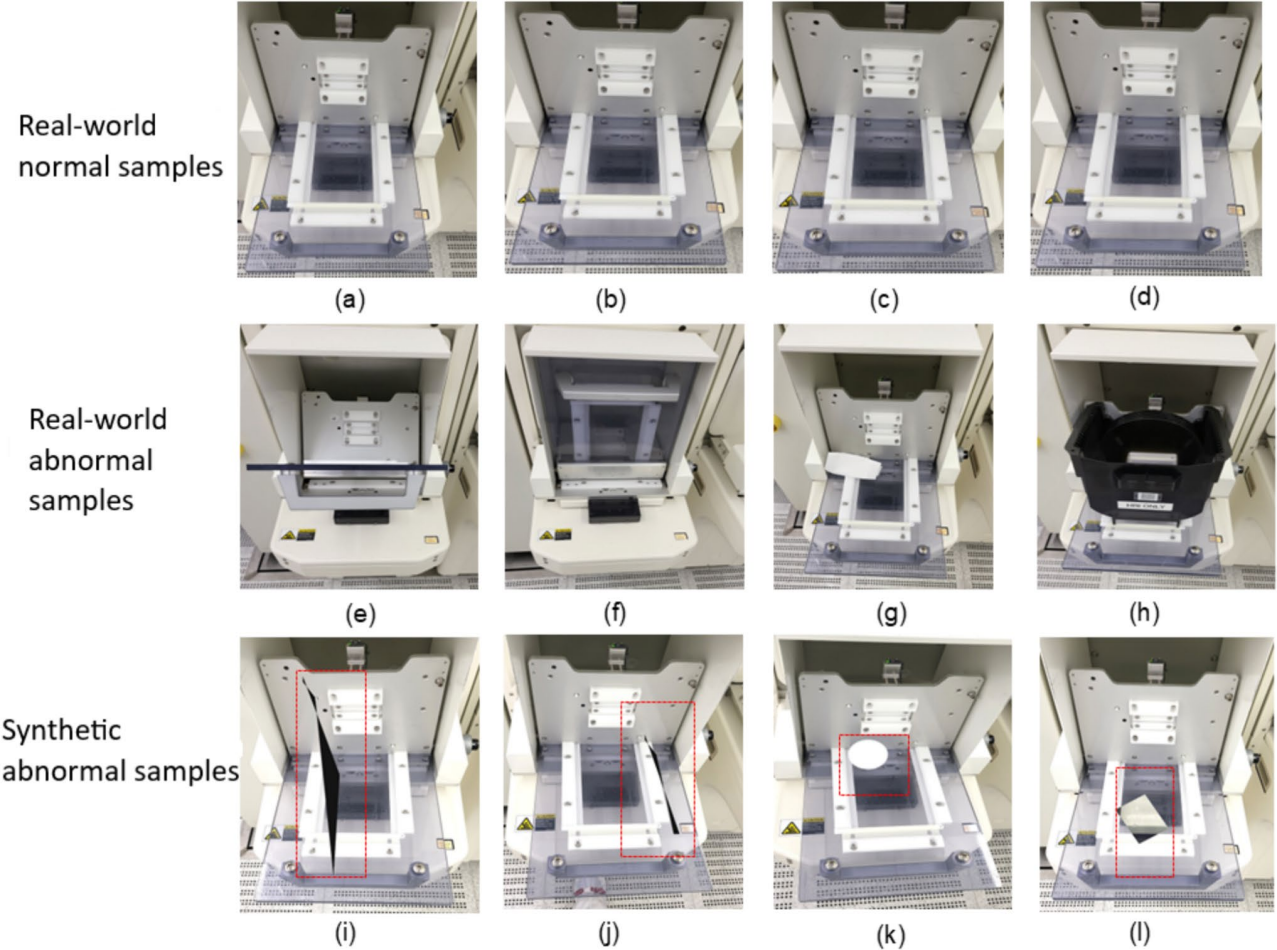
Illustration of Load Station (LS) samples during production, where samples (**a–d**) represent *Normal* Load Station state, (**e–h**) depict real *Abnormal* Load Station states. Specifically, (**e–f**) illustrate misalignment of the Load Station, where the Load Station is incorrectly positioned to receive a cassette for inspection. Figures (**g–h**) represent scenarios where another object occupies the LS preventing cassette placement. The third row (**i–l**) presents 2D synthetic obstructions superimposed on the target area, used to simulate *Abnormal* situations to train the DNNs in the experiments that follow

This study addresses the Load Station inspection problem in semiconductor manufacturing to handle wafer cassette prior to inspection. During the HRI process, wafers stored in secure load boxes are transferred to PEEK cassettes, which are then placed on the Load Station for inspection. Some Load Station samples are shown in Fig. 2. Before this placement, the vision system must verify two conditions: Correct Alignment - the Load Station door has opened correctly and the station is properly positioned to receive a cassette; Absence of Obstructions - the Load Station surface is free from foreign objects such as paper, tools, or other materials that would prevent correct cassette placement.

The primary contributions of this work can be expressed as:

- The study examines how training data size affects deep learning-based Load Station inspection accuracy in a semiconductor manufacturing context. It provides guidelines for researchers to deploy such inspection systems in data-scarce environments. This addresses a gap in the literature where systematic analysis of performance scaling with training data size is often absent.
- The applied approach is validated on operational semiconductor manufacturing data samples, unlike studies using simulated or laboratory environments. This real-world validation demonstrates the practical viability of deep learning inspection with limited training data.
- A comparison of YOLOv5 and YOLOv8 architectures across multiple model sizes (nano to extra-large), evaluating detection accuracy and data efficiency across model capacities to guide industrial deployment decisions. The results show that YOLOv8n achieves the best data efficiency, reaching *0.981 ± 0.014* inspection accuracy and *0.842 ± 0.050* mAP@0.5 (mean ± std under 5-fold cross-validation) with only 40 samples per class (*T*-40), while YOLOv5 requires substantially more training data for comparable performance.
- The study provides recommendations for minimum training data requirements, model selection, and deployment considerations based on experimental findings. It concludes that reliable inspection can be achieved with as few as 40 labelled samples per class using transfer learning, with lightweight models like YOLOv8 variants (n, s) being preferable for data-scarce industrial applications.
- The study uses a hybrid dataset combining real Normal samples with synthetically generated obstruction samples to train robust models. This hybrid approach addresses the challenge of rare Abnormal conditions in operational environments and successfully allows the models to generalise to real Abnormal conditions despite being trained primarily on synthetic obstructions.

These contributions address a critical gap in the literature: while deep learning methods for manufacturing inspection are well-established, researchers lack guidance on deployment under realistic data constraints. Existing studies on deep learning-based manufacturing inspection typically employ whatever training data are available and report results for a single configuration. As shown in Table 1, systematic analysis of how performance scales with training data size is notably absent from the literature. The challenge in this paper, for smart manufacturing facilities, is the lack of real data to support the deployment of accurate deep learning based solution.

**Table 1:** Summary of deep learning approaches for industrial inspection and quality control in manufacturing. The studies are categorised by application domain, methodology, and key contributions, highlighting the gap in data requirement analysis

Study	Application	Method	Key Contribution	Limitation
*Object Detection and Visual Inspection*				
[25]	Surface morphology (DED)	YOLOv7 + TL	Effective inspection with 240 samples using transfer learning	Single config; no data-size analysis
[8]	Metallic defects (WAAM)	YOLOv4	Real-time defect detection in additive manufacturing	Fixed dataset; limited defect types
[4]	Tool wear (broaching)	ML + signal processing	Reduced errors through automated monitoring	Requires sensor data; not vision-based
*Synthetic Data and Domain Adaptation*				
[26]	Assembly inspection	Faster R-CNN + CAD	Synthetic CAD data can substitute real images	Domain gap (synthetic vs real)
[7]	Construction	Hybrid DNN	Combined synthetic and real images	Single training config
[19]	Segmentation	Domain adaptation	Addressed domain shift from synthetic data	Segmentation only
*Anomaly Detection in Smart Manufacturing*				
[2]	Robotic anomaly	Score-based	Detected anomalies from learned trajectories	Requires continuous data
[1]	Anomaly benchmark	Unsupervised	Established MVTec AD benchmark	Fixed benchmark
[3]	Hot-spot defects (L-PBF)	Video classification	Real-time in-situ monitoring	Requires high-speed video
*Digital Twin and System Integration*				
[12]	Flexible manufacturing	Digital twin	DT integration for manufacturing intelligence	Simulation only
*Few-Shot and Data-Efficient Learning*				
[11]	Metal surface defects	Few-shot learning	Classification with 5–20 samples per class	Classification only

The remainder of this paper is organised as follows: Section 2 reviews related work on deep learning in smart manufacturing and visual inspection systems. Section 3 describes the experimental design and methodology. Section 4 details the model training and evaluation metrics. Section 5 presents experimental results examining the effect of training data size on inspection accuracy. Section 6 discusses the findings and their implications for industrial deployment and Section 7 concludes the paper with future research directions.

## Related work

The application of deep learning in manufacturing has grown significantly with the advent of Industry 4.0. [2] proposed a score-based anomaly detection framework for smart manufacturing systems where robots learn task execution from human demonstrations. Their work addresses the challenge of detecting when a robot enters states that are significantly different from those learned during model training, which is related to our goal to detect *Abnormal* load station conditions during production. Recent research for tool inspection demonstrates the importance of reliable results in manufacturing. [4] applied a machine learning based solution for a tool wear monitoring system for high-speed broaching processes to reduce production errors. This study indicates how automated monitoring systems can significantly reduce manufacturing defects and improve quality control in industrial settings. Digital twin technologies have also emerged as a complementary approach to physical inspection systems. [12] presented a framework to conceive digital twins in flexible manufacturing systems; the applied approach demonstrated how virtual representations can enhance manufacturing intelligence. While digital twins provide valuable simulation capabilities, physical inspection systems remain essential to verify the actual state of equipment.

Object detection, often used for industrial inspection, uses deep neural networks and has two main categories. Single-stage detectors, such as the YOLO family [5, 6, 17] and SSD [10], process images in a single forward pass, enabling real-time inference. Two-stage detectors, such as Faster R-CNN [18] and DetectoRS [15], achieve higher accuracy however with greater computational cost. For industrial applications requiring real-time performance, single-stage detectors are generally preferred. [25] applied YOLOv7 with transfer learning for surface morphology inspection in directed energy deposition, using only 240 training samples. [8] used YOLOv4 for automatic fault identification in wire and arc additive manufacturing. These studies demonstrate the viability of YOLO-based approaches for manufacturing inspection however do not systematically examine minimum data requirements.

The challenge of limited training data is well-recognised in industrial machine learning. Transfer learning is often considered as a foundational solution to limited training data by leveraging pre-trained models [20]. Models pre-trained on large datasets like COCO [9] can be subsequently fine-tuned with domain-specific data, reducing the high-volume of task-specific training data required.

Synthetic data generation has been explored as a means of augmenting limited real data. Sankaranarayanan et al. [19] demonstrated that combinations of synthetic and real data can improve model generalisation. Kim et al. [7] showed that hybrid training with synthetic and real construction images can overcome data shortages. Zhu et al. [26] used synthetic CAD data for assembly quality inspection, achieving good results with limited real training samples. Beyond detection, benchmark datasets such as MVTec AD [1] have standardised the unsupervised evaluation of anomaly detection, and video-based classification has enabled real-time monitoring of process defects [3]; both, however, target evaluation settings distinct from the supervised, data-scarce detection task considered here.

Few-shot learning has emerged as a crucial paradigm for data-scarce industrial inspection. [11] proposed attention-guided few-shot learning for metal surface defect classification with as few as 5–20 samples per class, demonstrating that meta-learning can substantially reduce data requirements compared to standard transfer learning. Our approach differs in two key aspects: first, we address object detection (localisation and classification), which is more demanding than classification alone; second, rather than relying on meta-learning, we combine transfer learning with synthetic data generation, which is more practical for industrial deployment because it does not require a base set of related tasks for meta-training.

Data augmentation techniques include geometric transformations, colour adjustments, and Gaussian noise as standard practices to expand limited datasets [14]. Despite these advances, a limitation exists in the literature regarding systematic guidance on minimum data requirements for industrial inspection deployment. Existing studies typically report results for a single training set size without examining how performance scales with data availability. Our study addresses this issue by providing empirical evidence across multiple training set sizes for industrial inspection. While deep learning has proven effective for manufacturing inspection, researchers lack clear guidance on how much training data is sufficient for reliable deployment. Notably, none of these studies systematically examines how performance scales with training data availability. Most studies use whatever data are available without systematically examining data requirements. This is problematic for manufacturing facilities where data collection is costly, and experts need evidence-based guidelines for project planning. Our study addresses this gap by examining the relationship between training data availability and model learning ability, leading to the evaluation of model architectures and their performance to enable specific deployment recommendations.

## Experimental design

**Fig. 3:**
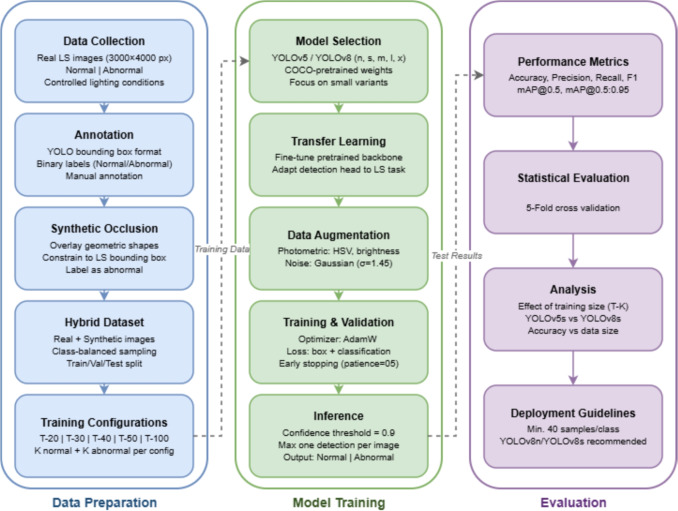
Overall framework for Load Station inspection: The *Data Preparation* stage prepares hybrid datasets to combine real and synthetic *Abnormal* samples across five training configurations (*T*-20 to *T*-100). The *Model Training* stage applies transfer learning with COCO-pretrained YOLOv5 and YOLOv8 architectures. The *Evaluation* stage assesses performance on real test data and derives minimum data requirements for industrial deployment

The overall methodology, from data acquisition to model deployment, is illustrated in Fig. 3. Details of these steps are explained in the following sections.

### Problem formulation

Let $D_{\text {real}}$ denote the complete set of industrial Load Station images collected from the manufacturing environment. Each image $I \in D_{\text {real}}$ is labelled as either *Normal* (N) or *Abnormal* (A), where *Normal* (N) belongs to the state where the Load Station door is fully open, the station is correctly aligned, and the Load Station surface is free of obstructions. The *Abnormal* class comprises three sub-categories: $\mathcal {A}_1$: Cassette present on the LS, preventing placement of a new cassette; $\mathcal {A}_2$: Foreign objects on the Load Station surface including tissue, tools, and debris; and $\mathcal {A}_3$: Load Station misalignment where the door is not fully open or the station is incorrectly positioned.

We collapse $\mathcal {A}_1$, $\mathcal {A}_2$ and $\mathcal {A}_3$ into a single *Abnormal* class, yielding a binary classification problem:

$$\text {Load Station state} \in \{\text {\textit{Normal}}, \text {\textit{Abnormal}}\}.$$

The primary objective is to train a detector model *M* to accurately classify the Load Station state using image data while minimising the number of training samples required for reliable deployment in a real manufacturing environment.

### Data acquisition

Data were collected from an operational semiconductor manufacturing facility. The samples were captured with a resolution of *3000 × 4000* pixels under controlled industrial lighting conditions and from different angles and distances. The complete real dataset $D_{\text {real}}$ comprises 540 RGB images: 313 *Normal* ($\mathcal {N}$) samples and 227 *Abnormal* ($\mathcal {A}$) samples, distributed across $\mathcal {A}_1$, $\mathcal {A}_2$ and $\mathcal {A}_3$. Specifically, the 227 *Abnormal* samples consist of 55 $\mathcal {A}_1$ (cassette present), 27 $\mathcal {A}_2$ (foreign object), and 145 $\mathcal {A}_3$ (misaligned) images. All samples were manually annotated using bounding boxes in YOLO format to indicate the Load Station region. An unseen subset of real samples is reserved as the *test set* and *validation set*.

### Synthetic obstructed samples generation

**Fig. 4:**
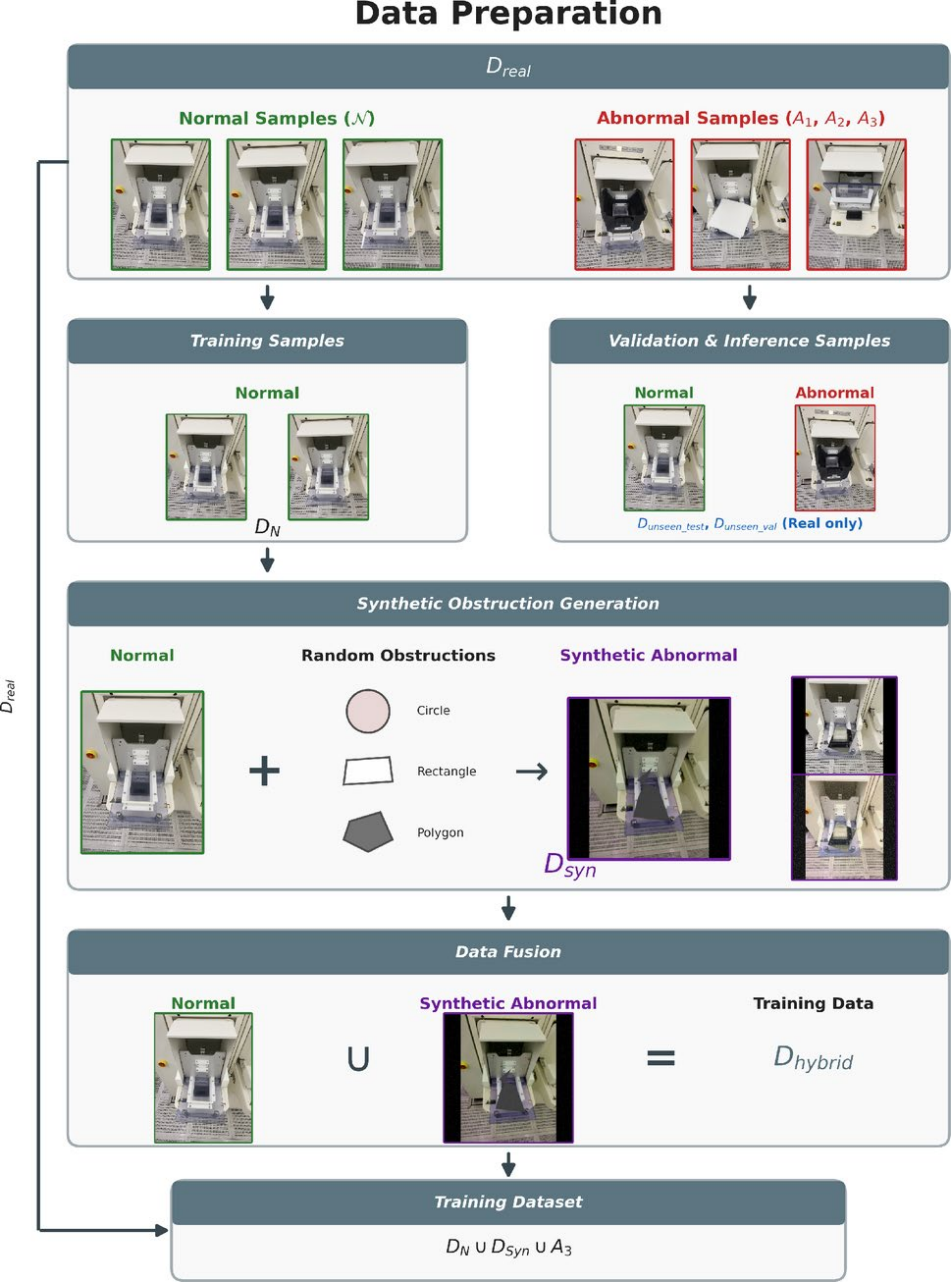
Overview of Load Station (LS) data preparation for model training, validation, and inference. The training dataset consists of real *Normal* images and synthetic *Abnormal* images, while the validation and inference datasets contain real target images

In real manufacturing environments, some *Abnormal* conditions, particularly severe obstructions of the Load Station surface, are rare and difficult to capture systematically. Intentionally placing foreign objects on the Load Station to collect data would disrupt production and may violate safety and contamination-control procedures. As a result, the number of available real obstructed examples is limited compared to *Normal* samples. To address this imbalance and enrich the *Abnormal* class under data scarcity, we generate *synthetic obstructed* $\mathcal {A}_s$ Load Station images from real *Normal* images. The pipeline to generate $\mathcal {D}_{\text {syn}}$ is illustrated in Fig. 4. The goal is not to introduce a novel synthetic data generation method, rather it is to provide the models with a broader range of plausible obstruction patterns for training, thus retaining the real data for the validation and testing.

A subset of *Normal* Load Station images is selected, and their existing bounding box annotations are used to locate the Load Station region. Within this region, simple geometric shapes (rectangles, circles and polygons) are overlaid at random positions and sizes, whilst being constrained to lie inside the Load Station bounding box. The background outside the Load Station region is left unchanged. This procedure produces images with synthetic augmentations in which the Load Station surface appears partially or fully obstructed, while preserving the original camera viewpoint, lighting and background context of the real factory environment. Each of these images is labelled as *Abnormal* and they inherit the Load Station bounding box from the corresponding *Normal* image. Prior to training, we prepare $\mathcal {D}_{\text {hybrid}}$ where we combine *Normal* $\mathcal {N}$ and *Synthetic* $\mathcal {D}_{\text {syn}}$. We also include some samples of misaligned Load Stations $\mathcal {A}_3$ within $\mathcal {D}_{\text {hybrid}}$. The details of the training configuration are summarised in Table 2. For validation and testing sets, we used the real-world samples $\mathcal {N}$, $\mathcal {A}_1$, $\mathcal {A}_2$ and $\mathcal {A}_3$.

**Table 2:** Sample distribution per training configuration. *Normal* ($\mathcal {N}$) samples are real; misaligned ($\mathcal {A}_3$) samples are real *Abnormal* images; obstructed ($\mathcal {D}_{\text {syn}}$) samples are synthetic *Abnormal* images generated from *Normal* images

Config.	Real $\mathcal {N}$	Obstructed $\mathcal {D}_{\text {syn}}$	Misaligned $\mathcal {A}_3$
*T*-20	20	15	5
*T*-30	30	20	10
*T*-40	40	25	15
*T*-50	50	30	20
*T*-100	100	75	25

### Training set configurations

To investigate the relationship between training data size and inspection accuracy under realistic data limitations, we design experiments with five training configurations: *T*-20, *T*-30, *T*-40, *T*-50, and *T*-100. Configuration *T*-*K* corresponds to a training set containing *K* *Normal* and *K* *Abnormal* samples. The *Abnormal* class in each configuration is a hybrid of real misaligned and synthetic obstructed samples $\mathcal {A}_s$. The sample distribution per configuration is summarised in Table 2.

For very small training sets (*T*-20 to *T*-50), the number of misaligned samples is gradually increased (from 5 to 20), and the remaining *Abnormal* samples in each configuration are filled with synthetic obstructed samples $\mathcal {A}_s$. For *T*-100, misaligned samples (25 images) are included, and the remaining samples are used for the validation and test sets. For each configuration, training samples are randomly selected from $D_{\text {real}}$ for both *Normal* and misaligned cases. In contrast, synthetic obstructed samples are chosen from generated samples, while maintaining the set sizes in Table 2. A separate validation set of real Load Station images is maintained across all configurations and is used for hyperparameter tuning and model selection. The final performance is evaluated on the fixed test set of 186 real images, which remains unseen during training and validation. For each training configuration, we perform 5-fold cross-validation by repartitioning the training pool into 5 disjoint folds; the held-out fold is used as the validation set and the remaining four are used for training. We further repeat each fold with three random initialisation seeds (1, 42, 2024) for YOLOv8s and one seed (42) for YOLOv8n, yielding 15 and 5 trained models per configuration respectively. The fixed test set of 186 real images is held constant across all folds and seeds, ensuring a consistent benchmark.

### Image preprocessing

The industrial samples are captured at *3000 × 4000* pixels, which exceeds the input resolution expected by the YOLO architectures. All images are preprocessed using the letterbox transformation: each image is resized while preserving its aspect ratio so that the longer side matches 640 pixels, and the remaining region is padded with a constant grey value (114, 114, 114) to produce a *640 × 640* input. Bounding-box annotations are rescaled by the same factor and translated to account for the padding, so the labels remain aligned with the Load Station region after resizing. The same preprocessing is applied to training, validation, and test images. The Load Station occupies 40–60% of each captured frame, so the region of interest remains well-represented at *640 × 640*. The visual cues relevant to inspection (obstructions on the surface and door misalignment) are coarse spatial features rather than fine-grained textures and remain distinguishable at the reduced resolution. The cross-validated performance in Section 5.1 confirms that the task-relevant information is preserved.

### Data augmentation

After preparation of the training set configurations, standard image augmentation techniques are applied with controlled values to further improve model generalisation ability. Table 3 shows the sample configuration after augmentation. The augmentation pipeline combines geometric and photometric transformations applied with controlled probabilities, producing approximately three augmented variants per source image. First, geometric augmentations such as horizontal flip and small in-plane rotations are applied to increase robustness to viewpoint variation around the Load Station. Vertical flips are not applied as upside-down Load Stations do not occur in operational settings.

The greyscale transformation is used to reduce sensitivity to colour variations and focus the model on structural cues within the Load Station region. A rotation by a small angle *θ* is applied due to the Load Station’s strict alignment for operational state, as

1 $$\begin{aligned} O_i(x, y) = I_i\bigl (\cos (\theta ) \cdot x - \sin (\theta ) \cdot y,\ \sin (\theta ) \cdot x + \cos (\theta ) \cdot y\bigr ) \end{aligned}$$

where *θ* is sampled from $[-15^\circ , 15^\circ ]$.

**Table 3:** Balanced-sample configuration for model training after geometric augmentation. The *Abnormal* class contains synthetic obstructions and real misaligned Load Station samples

Configuration	Normal	Abnormal
*T*-20	60	60
*T*-30	90	90
*T*-40	120	120
*T*-50	150	150
*T*-100	300	300

To handle illumination variations in the real environment, exposure and colour properties are randomly applied. Brightness (*b*) is varied by $\pm 10\%$ relative to the original pixel intensity values, and hue (*h*) is shifted by up to $\pm 25^\circ$ in the HSV colour space. The combined effect of hue and brightness adjustment is modelled as:

2 $$\begin{aligned} O_i(x, y) = \mathcal {H}(I_i(x, y),\ h) + b \end{aligned}$$

where $\mathcal {H}(\cdot , h)$ denotes a hue transformation applied to the input image $I_i(x, y)$.

Finally, to enhance robustness against blur and camera-focus variations, random Gaussian noise is added to the training samples:

3 $$\begin{aligned} O_i(x, y) = I_i(x, y) + \alpha \cdot G_s(x, y) \end{aligned}$$

where $G_s(x, y)$ is zero-mean Gaussian noise with standard deviation $\sigma _s = 0.05$, and *α = 1.45* scales the noise amplitude (as in Table 4). Augmentations are applied only to the training data; validation and test images are kept in their original form in the held-out set. The augmented dataset sizes shown in Table 3 reflect the cumulative output after applying the augmentation pipeline three times per source image. Application probabilities and parameter ranges are summarised in Table 4. These augmentations expand the effective training dataset and reduce overfitting.

**Table 4:** Augmentation pipeline parameters. Each transformation is applied independently with the specified probability per source samples, with parameters drawn uniformly from the specified range

Transformation	Probability	Parameter range
Horizontal flip	0.50	—
Rotation	0.50	$\theta \in [-15^\circ ,\,+15^\circ ]$
Brightness shift	0.50	$b \in [-10\%,\,+10\%]$
Hue shift	0.50	$h \in [-25^\circ ,\,+25^\circ ]$
Greyscale	0.20	—
Gaussian noise	0.30	*α = 1.45*, $\sigma _s = 0.05$

## Model training

We evaluated one-stage object detectors from the YOLOv5 and YOLOv8 families. The detailed cross-validation analysis focuses on YOLOv8n and YOLOv8s, with YOLOv5 variants and larger YOLOv8 variants (m, l, x) reported for architecture comparison in Section 5. YOLOv5s is a small variant of the YOLOv5 family with approximately 7 M parameters, offering a practical trade-off between accuracy and inference speed and providing a lightweight baseline suitable for real-time deployment on industrial hardware. YOLOv8s is a small YOLO variant with architectural improvements, including a revised backbone and detection head. In many vision tasks, YOLOv8s achieves higher accuracy than YOLOv5s, typically at a slightly higher computational cost.

All models are initialised with COCO-pretrained weights and fine-tuned on the Load Station training configurations described in Section 3. The cross-validated study uses YOLOv8n and YOLOv8s (the lightweight variants suitable for industrial deployment), each trained over 5 cross-validation folds. Larger YOLOv8 variants (m, l, x) and YOLOv5 variants are also trained to explore the speed–accuracy trade-off in the architecture comparison. The parameter configuration details for these models are provided in Table 5.

**Table 5:** Training hyperparameters and protocol. The same configuration is used across all experiments unless otherwise stated

Hyperparameter	Value	Notes
*Architecture and initialisation*		
Detector families	YOLOv5, YOLOv8	Cross-validation focuses on YOLOv8n and YOLOv8s
Pre-trained weights	COCO	Transfer learning starting point
Input resolution	*640 × 640*	Letterbox resize
*Optimisation*		
Optimiser	AdamW	Used for all reported runs
Initial learning rate	$1.0 \times 10^{-3}$	Default Ultralytics schedule
Momentum / $\beta _1$	0.937	SGD momentum / Adam $\beta _1$
Weight decay	$5.0 \times 10^{-4}$	L2 regularisation
Batch size	16	Reduced to 8 for larger variants
Epochs	50	Full schedule run
Loss function	box + cls + DFL	Standard YOLOv8 multi-task loss
*Cross-validation protocol*		
CV folds	5	Stratified by Normal / Abnormal
Random seeds (YOLOv8s)	{1, 42, 2024}	15 runs per training configuration
Random seeds (YOLOv8n)	{42}	5 runs per training configuration
Test set	186 real images	Frozen across all folds and seeds
*Inference*		
Confidence threshold	0.9	Conservative; suited to quality-critical inspection
IoU threshold (NMS)	0.7	Default Ultralytics NMS
Max detections per image	1	One Load Station decision per image

### Evaluation metrics

The training set configuration is designed to ensure the model’s learning ability with limited data, whilst being sufficient to handle realistic changes in the factory environment. We evaluate model performance to measure how well the DNNs have learned discriminative features for Load Station inspection and to identify the best-performing architecture.

**Fig. 5:**
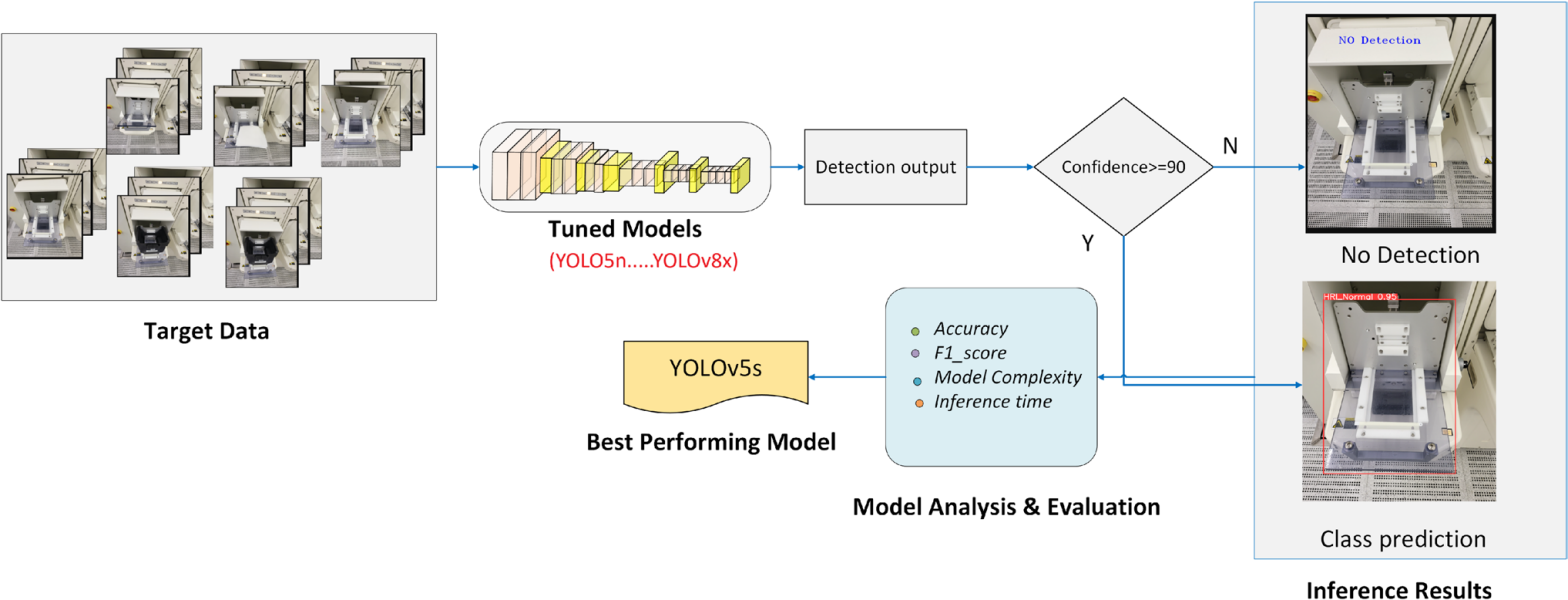
Illustration of the inference and performance evaluation process. Test images are processed by the trained models, and the detection outputs are filtered using a fixed confidence threshold. Load Station instances are then classified as *Normal* or *Abnormal*, or marked as *No Detection* if the confidence is below the threshold. The final decisions are evaluated using metrics such as accuracy, *precision*, *recall*, and *F1-score* to identify the most suitable model for deployment

The testing data are separated from the training data after preprocessing, as illustrated in the pipeline in Fig. 5. The trained model processes the target images and produces detection outputs, which are filtered using a confidence threshold. We set *τ = 0.9* as a conservative deployment threshold appropriate for quality-critical industrial applications, where high-confidence detections are preferred to minimise false positives. The trade-off this imposes on recall is reported and discussed in Section 5. Based on this threshold, Load Station instances are either classified or discarded.

During training and validation, we follow the standard YOLO evaluation metrics, including precision (*P*), recall (*R*), mean Average Precision at 0.5 IoU threshold (*mAP@0.5*), and mean Average Precision across IoU thresholds from 0.5 to 0.95 (*mAP@0.5:0.95*). These metrics are recorded at each epoch and plotted as learning curves to analyse the models’ convergence and generalisation behaviour.

For inference time evaluation on the unseen real test set, we use a fixed confidence threshold of 90%. If the highest-confidence detection in an image is below this threshold, the image is labelled as *No Detection*. As shown in Fig. 5, the final decision for each image is therefore one of three options: *No Detection*, *Normal*, or *Abnormal*. We subsequently reclassify cases where no detection occurs as *Abnormal* under our fail-safe protocol. To simplify the decision process and reflect the operational requirement of a single Load Station decision per image, we restrict the system to at most one detection per image at inference time, keeping only the bounding box with the highest confidence and discarding any others.

After applying these rules, the model performance is quantified using *Accuracy*, *Precision*, *Recall* and *F1-score*, defined as:

4 $$\begin{aligned} \text {\textit{Accuracy}} = \frac{\text {TP} + \text {TN}}{\text {TP} + \text {TN} + \text {FP} + \text {FN}}, \end{aligned}$$

5 $$\begin{aligned} \text {\textit{Precision}} = \frac{\text {TP}}{\text {TP} + \text {FP}}, \end{aligned}$$

6 $$\begin{aligned} \text {\textit{Recall}} = \frac{\text {TP}}{\text {TP} + \text {FN}}, \end{aligned}$$

7 $$\begin{aligned} \text {\textit{F1-score}} = \frac{2 \cdot \text {\textit{Precision}} \cdot \text {\textit{Recall}}}{\text {\textit{Precision}} + \text {\textit{Recall}}}. \end{aligned}$$

Here, TP (True Positive) denotes *Abnormal* samples correctly classified as *Abnormal*, including cases where no detection occurs (these are classified as *Abnormal* under the fail-safe protocol). TN (True Negative) denotes *Normal* samples correctly classified as *Normal*. FP (False Positive) denotes *Normal* samples incorrectly classified as *Abnormal*, including *Normal* samples with no detection. FN (False Negative) denotes *Abnormal* samples incorrectly classified as *Normal*.

## Experimental results

All experiments were conducted on a system equipped with an NVIDIA RTX 3070 GPU, a 12th Gen Intel Core processor, and 32 GB RAM. The software environment consisted of Python 3.10, PyTorch 2.3.1, and Torchvision 0.18.1 run on Ubuntu 22.04.3. Both YOLOv5 and YOLOv8 model variants were initialised with COCO pretrained weights and fine-tuned on the Load Station training set using the configurations described in Section 4.

The primary objective of this work is to determine how many labelled training samples are needed for reliable Load Station inspection. To answer this question, we evaluated model performance across five training configurations (*T*-20, *T*-30, *T*-40, *T*-50, and *T*-100), where *T*-*K* denotes a training set containing *K* samples of each class (*Normal* and *Abnormal*). For statistical robustness, every training configuration is evaluated under 5-fold cross-validation, with the YOLOv8s configurations additionally repeated across three random initialisation seeds (1, 42, 2024), giving 15 trained models per configuration; YOLOv8n is run for 5 folds with seed 42, giving 5 trained models per configuration. The 186-image test set is held constant across all folds and seeds. All test-set results reported in this section use the inference protocol described in Section 4.1 (confidence threshold *τ = 0.9*, single detection per image).

### Test set evaluation

**Fig. 6:**
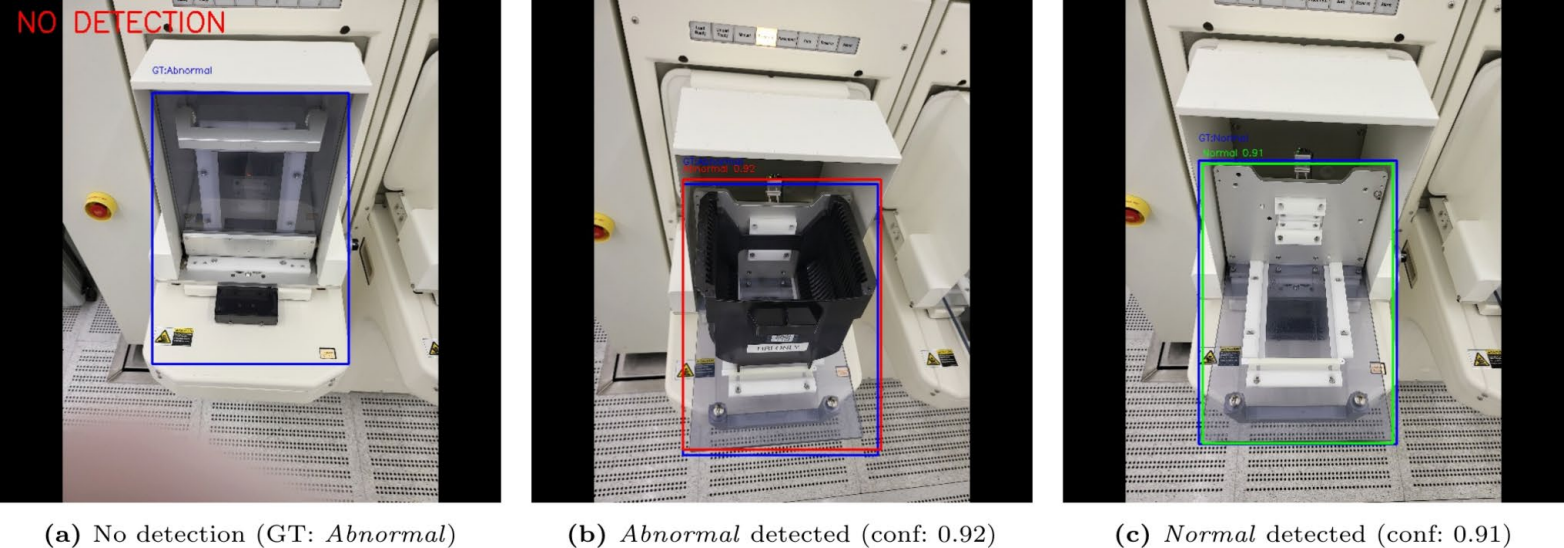
Sample inference results from the test set. Blue boxes indicate ground truth annotations, green boxes show *Normal* detections, and red boxes show *Abnormal* detections. (**a**) An *Abnormal* case with no detection above the confidence threshold, correctly classified as *Abnormal*. (**b**) Correct *Abnormal* detection. (**c**) Correct *Normal* detection

Figure 6 shows representative inference outputs from the test set using YOLOv8s trained on *T*-40. The model produces detection outputs with associated confidence scores. Images with confidence *\ge 0.9* are classified as either *Normal* or *Abnormal*, while images with confidence below this threshold are labelled as *No Detection*. In the examples shown, the model correctly identifies a *Normal* state with high confidence (0.91) and detects an *Abnormal* condition with a confidence score of 0.92.

Table 6 presents the full cross-validation test-set performance of YOLOv8n and YOLOv8s across all five training configurations. Several findings emerge: at T-40, YOLOv8n achieves accuracy and mAP@0.5; at T-50 it reaches its peak of accuracy and mAP@0.5. YOLOv8s reaches accuracy values in the same range, however consistently shows lower Recall and F1, indicating that the smaller-capacity nano variant generalises better on this limited-data task.

**Table 6:** Cross-validation test-set performance of the proposed method across training sizes. All values are mean ± std over folds and seeds

Model	T-K	Accuracy	Precision	Recall	F_1_	mAP@0.5
YOLOv8n	T-20	0.969 ± 0.019	1.000 ± 0.000	0.657 ± 0.152	0.782 ± 0.116	0.858 ± 0.036
YOLOv8s	T-20	0.964 ± 0.018	0.933 ± 0.249	0.638 ± 0.253	0.742 ± 0.242	0.807 ± 0.108
YOLOv8n	T-30	0.969 ± 0.002	1.000 ± 0.000	0.554 ± 0.261	0.673 ± 0.240	0.781 ± 0.103
YOLOv8s	T-30	0.965 ± 0.017	0.933 ± 0.249	0.456 ± 0.317	0.557 ± 0.327	0.847 ± 0.074
YOLOv8n	T-40	0.981 ± 0.014	0.999 ± 0.002	0.700 ± 0.235	0.800 ± 0.167	0.842 ± 0.050
YOLOv8s	T-40	0.980 ± 0.014	0.933 ± 0.249	0.430 ± 0.197	0.569 ± 0.239	0.809 ± 0.101
YOLOv8n	T-50	0.993 ± 0.008	1.000 ± 0.000	0.750 ± 0.133	0.850 ± 0.087	0.946 ± 0.047
YOLOv8s	T-50	0.982 ± 0.016	1.000 ± 0.000	0.516 ± 0.167	0.663 ± 0.158	0.873 ± 0.076
YOLOv8n	T-100	0.986 ± 0.015	1.000 ± 0.000	0.683 ± 0.160	0.801 ± 0.119	0.912 ± 0.100
YOLOv8s	T-100	0.977 ± 0.015	1.000 ± 0.000	0.477 ± 0.174	0.626 ± 0.173	0.880 ± 0.097

Performance improves with training size, however with diminishing returns. The *mAP@0.5* for YOLOv8n rises from 0.858 at *T*-20 to 0.946 at *T*-50, then drops slightly at *T*-100 (0.912). This behaviour reflects the fact that the misaligned ($\mathcal {A}_3$) sample budget grows from 5 to 25 images across the configurations and saturates at *T*-50, while the synthetic obstruction count continues to expand. Larger training sizes therefore add redundant variation rather than new failure-mode coverage. Precision values approach 1.0 across all configurations because the models are trained on real samples from the *Normal* class and hence learn reliably. *Recall* (or equivalently F_1_) is therefore the more discriminating metric on this benchmark; we report all four metrics for completeness.

Several configurations exhibit large standard deviations on Recall (e.g. YOLOv8s at *T*-30, *0.456 ± 0.317*). Standard-deviation magnitude itself is therefore informative and is included in every reported value.

### Convergence and overfitting analysis

**Fig. 7:**
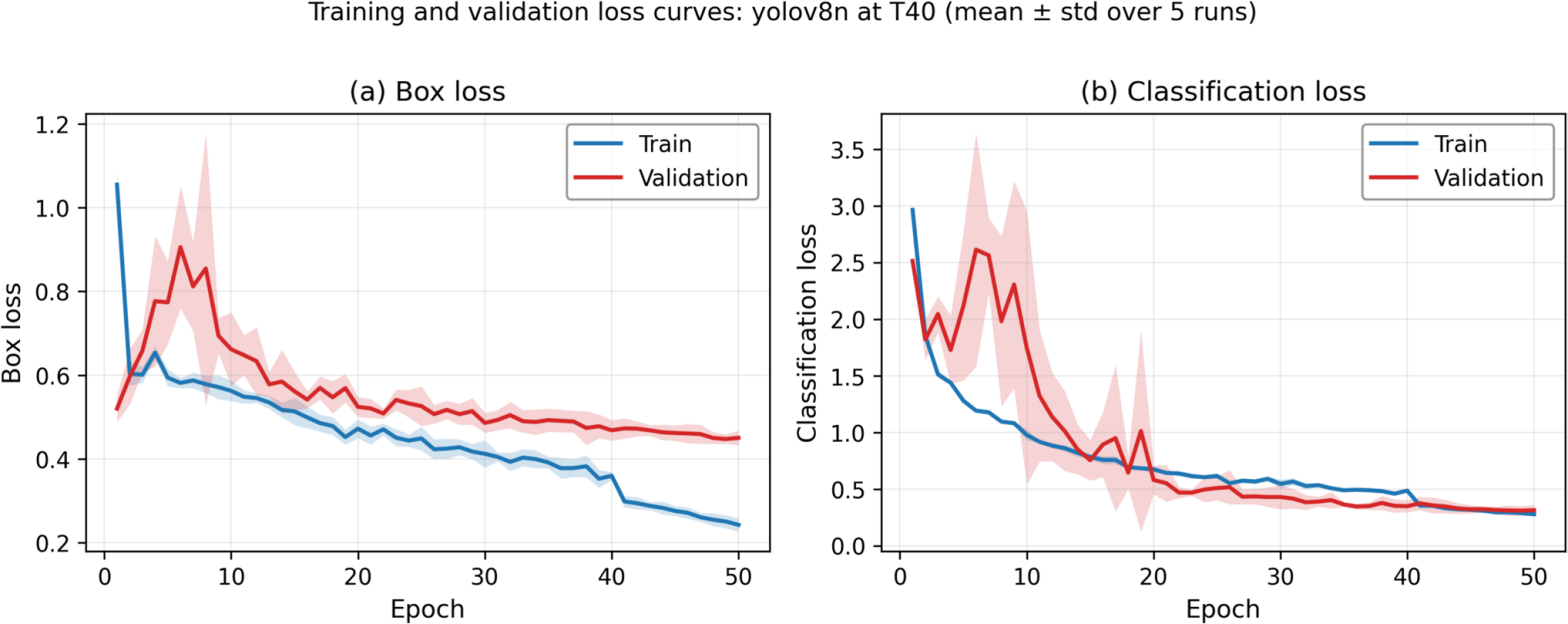
Training and validation loss curves for YOLOv8n at *T*-40. (**a**) Box regression loss, (**b**) Classification loss. Solid lines show mean values across 5 cross-validation folds; shaded bands represent *± 1* standard deviation

Figure 7 shows training and validation loss curves for YOLOv8n at *T*-40, aggregated across the 5 cross-validation folds. Both box and classification losses decrease on the training set and converge to stable plateaus by approximately epoch 40. The validation curves follow the same trend without divergence, indicating that 50 epochs are sufficient for convergence and that the hybrid training strategy does not induce substantial overfitting. A moderate residual gap remains between train and validation box loss (approximately 0.20 at convergence), consistent with expectations for limited-data transfer learning, while classification loss converges to nearly the same value on both sets. The narrow standard-deviation bands on the training curves indicate reproducibility across folds; the wider bands on the validation curve during early epochs reflect the limited size of each fold’s validation set.

### Ablation study

To isolate the contribution of synthetic obstructions and real misaligned samples to overall performance, we conducted an ablation study at *T*-40, the configuration that the validation analysis identifies as a practical operating point.

**Fig. 8:**
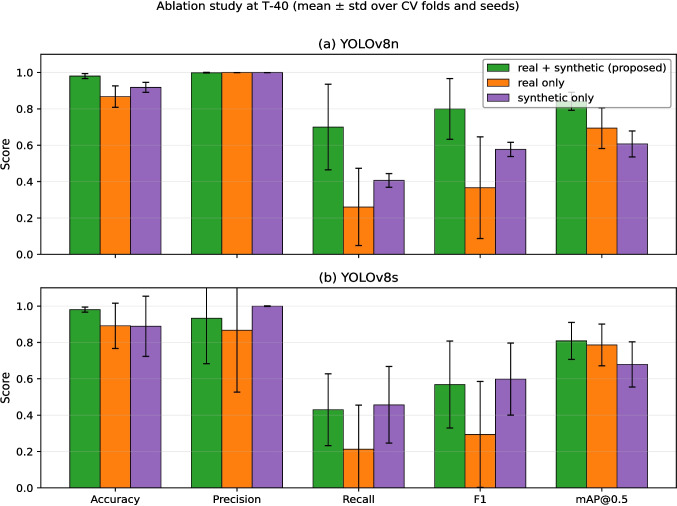
Ablation study at *T*-40: (**a**) YOLOv8n and (**b**) YOLOv8s. Bars compare the proposed hybrid (real + synthetic) against *real-only* (no synthetic obstructions) and *synthetic-only* (no real $\mathcal {A}_3$). *Normal* samples are real in all three regimes; only the *Abnormal* source varies. Error bars: *± 1* standard deviation across CV folds and seeds

Three training regimes are compared, each evaluated under 5-fold cross-validation with the same fixed test set: (i) the *proposed hybrid* training using real *Normal*, real $\mathcal {A}_3$, and synthetic obstructions; (ii) *real-only*, retaining the same real *Normal* samples however training on only the real $\mathcal {A}_3$ misaligned samples for the *Abnormal* class (no synthetic obstructions); and (iii) *synthetic-only*, retaining the same real *Normal* samples but training on only the synthetic obstructions for the *Abnormal* class (no real $\mathcal {A}_3$). *Normal* samples are real in every regime; the ablation isolates the contribution of each *Abnormal* data source. Results are summarised in Table 7 and visualised in Fig. 8.

**Table 7:** Ablation study at *T*-40, comparing the proposed hybrid training (real + synthetic) against *real-only* (no synthetic obstructions) and *synthetic-only* (no real $\mathcal {A}_3$). *Normal* samples are real in every regime; the ablation isolates the contribution of each *Abnormal* data source. All values are mean ± std over CV folds and seeds

Training Regime	Model	Accuracy	Precision	Recall	F_1_	mAP@0.5
Real + synthetic (proposed)	YOLOv8n	0.981 ± 0.014	0.999 ± 0.002	0.700 ± 0.235	0.800 ± 0.167	0.842 ± 0.050
	YOLOv8s	0.980 ± 0.014	0.933 ± 0.249	0.430 ± 0.197	0.569 ± 0.239	0.809 ± 0.101
Real only	YOLOv8n	0.868 ± 0.059	1.000 ± 0.000	0.260 ± 0.212	0.366 ± 0.280	0.694 ± 0.111
	YOLOv8s	0.891 ± 0.124	0.867 ± 0.340	0.213 ± 0.242	0.294 ± 0.291	0.786 ± 0.115
Synthetic only	YOLOv8n	0.918 ± 0.027	1.000 ± 0.000	0.407 ± 0.038	0.577 ± 0.039	0.607 ± 0.071
	YOLOv8s	0.889 ± 0.165	1.000 ± 0.002	0.457 ± 0.211	0.599 ± 0.199	0.679 ± 0.124

Removing either source of *Abnormal* variation harms performance substantially. For YOLOv8n, Recall drops from 0.700 in the proposed regime to 0.260 when synthetic obstructions are removed, and to 0.407 when real misaligned samples are removed. The corresponding F1 reductions are from 0.800 to 0.366 and 0.577 respectively. Although the accuracy values for the ablation regimes appear high (above 0.85), this is again a consequence of test-set imbalance rather than detection performance. The proposed hybrid combining both data sources is unambiguously the strongest regime on Recall, F1, and *mAP@0.5*, confirming that real and synthetic samples carry complementary training signal.

The aggregate ablation establishes that both data sources are essential; the next section addresses why they are complementary by decomposing the same ablation results by abnormal subclass.

### Per-subclass analysis

**Fig. 9:**
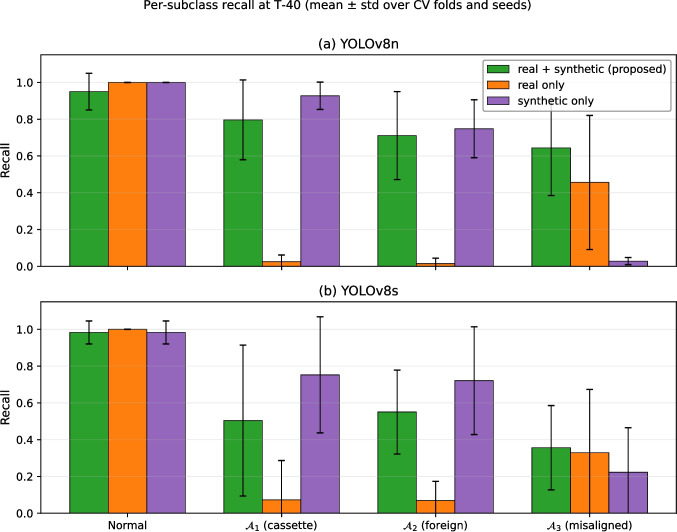
Per-subclass recall at *T*-40 across the three training regimes. (**a**) YOLOv8n and (**b**) YOLOv8s. The *Normal* column shows correct-reject rate on test-set Normal images; *Normal* training samples are identical (real) across all regimes. $\mathcal {A}_1$ (cassette), $\mathcal {A}_2$ (foreign object), $\mathcal {A}_3$ (misaligned door) columns show subclass-specific recall. Error bars: *± 1* standard deviation across CV folds and seeds

Building on the ablation in Section 5.3, we now decompose the same three training regimes by abnormal subclass to understand why the two data sources contribute complementary signal. The aggregated metrics in Section 5.3 conflate three distinct *Abnormal* subclasses with quite different visual characteristics. Table 8 and Fig. 9 report per-subclass recall at *T*-40 across the three training regimes.

**Table 8:** Per-subclass recall at *T*-40 across the three training regimes. Mean ± std over CV folds and seeds

Training Regime	Model	Normal	$\mathcal {A}_1$ (cassette)	$\mathcal {A}_2$ (foreign)	$\mathcal {A}_3$ (misaligned)
Real + synthetic (proposed)	YOLOv8n	0.950 ± 0.100	0.796 ± 0.217	0.711 ± 0.239	0.644 ± 0.259
	YOLOv8s	0.983 ± 0.062	0.504 ± 0.410	0.551 ± 0.228	0.357 ± 0.229
Real only	YOLOv8n	1.000 ± 0.000	0.025 ± 0.036	0.015 ± 0.030	0.456 ± 0.365
	YOLOv8s	1.000 ± 0.000	0.073 ± 0.214	0.069 ± 0.105	0.329 ± 0.344
Synthetic only	YOLOv8n	1.000 ± 0.000	0.927 ± 0.074	0.748 ± 0.158	0.028 ± 0.019
	YOLOv8s	0.983 ± 0.062	0.753 ± 0.316	0.721 ± 0.293	0.223 ± 0.243

For the proposed regime, YOLOv8n achieves recalls of 0.796, 0.711, and 0.644 on $\mathcal {A}_1$ (cassette present), $\mathcal {A}_2$ (foreign object), and $\mathcal {A}_3$ (misaligned door) respectively. The lower performance on $\mathcal {A}_3$ is informative: synthetic obstructions are generated by overlaying geometric shapes within the Load Station bounding box, which closely emulates the visual signature of cassette presence and foreign objects ($\mathcal {A}_1$ and $\mathcal {A}_2$). The misaligned door ($\mathcal {A}_3$), in contrast, is a structural change to the Load Station rather than an added object, and cannot be synthesised by the bounding-box-constrained obstruction generator. Training on $\mathcal {A}_3$ therefore relies entirely on the 15 real misaligned samples available per training fold.

The synthetic-only ablation makes this dependency explicit: without real $\mathcal {A}_3$ samples in training, recall on $\mathcal {A}_3$ collapses from 0.644 to 0.028 (YOLOv8n), while $\mathcal {A}_1$ and $\mathcal {A}_2$ recalls remain high (0.927 and 0.748). Conversely, the real-only ablation collapses $\mathcal {A}_1$ and $\mathcal {A}_2$ recalls (to 0.025 and 0.015) while leaving $\mathcal {A}_3$ recall at 0.456. This subclass-specific decomposition supports the design choice of combining real and synthetic data in proportion to the visual difficulty and availability of each subclass.

### Preliminary architecture screening

To select model variants for the cross-validated study, we first conducted a preliminary architecture screening across YOLOv5 and YOLOv8 variants from nano to extra large. Test-set accuracy and F_1_ scores from this 5-fold cross-validated screening (mean ± std over folds; YOLOv5s and YOLOv8s additionally averaged over three random seeds) are reported in Table 9.

**Table 9:** Architecture screening across YOLOv5 and YOLOv8 variants under 5-fold cross-validation. Test-set Accuracy and F_1_ at *τ =0.9*, reported as mean±std

	*T*-20		*T*-30		*T*-40		*T*-50		*T*-100	
Model	Acc	F_1_	Acc	F_1_	Acc	F_1_	Acc	F_1_	Acc	F_1_
YOLOv5n	0.963 ±0.012	0.876 ±0.072	0.971 ±0.012	0.636 ±0.119	0.976 ±0.013	0.784 ±0.038	0.989 ±0.012	0.642 ±0.311	0.977 ±0.010	0.679 ±0.084
YOLOv5s	0.971 ±0.014	0.688 ±0.221	0.976 ±0.011	0.424 ±0.239	0.977 ±0.011	0.507 ±0.185	0.984 ±0.014	0.469 ±0.197	0.979 ±0.013	0.526 ±0.178
YOLOv5m	0.966 ±0.024	0.552 ±0.320	0.974 ±0.018	0.751 ±0.139	0.979 ±0.014	0.602 ±0.202	0.981 ±0.014	0.542 ±0.099	0.977 ±0.012	0.628 ±0.161
YOLOv5l	0.982 ±0.024	0.703 ±0.358	0.990 ±0.013	0.764 ±0.206	0.983 ±0.013	0.637 ±0.336	0.986 ±0.015	0.612 ±0.201	0.979 ±0.009	0.773 ±0.091
YOLOv5x	0.993 ±0.013	0.363 ±0.448	0.985 ±0.012	0.537 ±0.262	0.985 ±0.012	0.546 ±0.087	0.987 ±0.012	0.460 ±0.307	0.977 ±0.010	0.732 ±0.068
YOLOv8n	0.969 ±0.019	0.782 ±0.116	0.969 ±0.002	0.673 ±0.240	0.981 ±0.014	0.800 ±0.167	0.993 ±0.008	0.850 ±0.087	0.986 ±0.015	0.801 ±0.119
YOLOv8s	0.964 ±0.018	0.742±0.242	0.965±0.017	0.557±0.327	0.980±0.014	0.569±0.239	0.982±0.016	0.663±0.158	0.977±0.015	0.626±0.173
YOLOv8m	0.959±0.022	0.786±0.109	0.971±0.011	0.701±0.179	0.979±0.015	0.790±0.050	0.975±0.009	0.552±0.233	0.976±0.021	0.750±0.092
YOLOv8l	0.975±0.021	0.757±0.261	0.983±0.013	0.701±0.354	0.985±0.012	0.725±0.096	0.987±0.008	0.681±0.106	0.982±0.011	0.776±0.070
YOLOv8x	0.988±0.024	0.628±0.321	0.971±0.007	0.767±0.187	0.969±0.025	0.866±0.155	0.981±0.006	0.648±0.159	0.983±0.012	0.869±0.089

Several observations from the screening guided the choice of YOLOv8n and YOLOv8s for the cross fold validation. First, accuracy saturates near 0.96–0.99 for every YOLO variant and training size for T20-T100, images with no confident detection are not penalised and the *Normal* class provided significant results as compared to Abnormal due to abundant class. This is the reason accuracy does not much fluctuate between architectures, and F_1_ together with its fold-to-fold variance is the more informative metric. Second, with respect to F_1_, the lightweight YOLOv8 variants (n, s) remain competitive compared with the much larger models while using an order of magnitude fewer parameters and FLOPs (YOLOv8x: 68 M parameters, 257 GFLOPs), whereas the YOLOv5 family and the larger YOLOv8 variants are less consistent across training sizes(T20-T100). Third, every variant exhibits substantial fold-to-fold variability: F_1_ standard deviations reach 0.30–0.45 for several configurations (YOLOv5x at *T*-20, *0.363 ± 0.448*; YOLOv5l at *T*-40, *0.637 ± 0.336*), while the lightweight YOLOv8 variants (particularly YOLOv8n) show comparatively narrow intervals.

Their combination of competitive F_1_ scores, low computational cost, and relative stability motivated their selection for the in-depth cross-validated analysis. The high variance observed even under cross-validation is itself an important finding: the wide F_1_ score intervals show that a point estimate from any single configuration can shift by 0.30–0.45, large enough to invert architecture rankings between training sizes. This underscores why the two lightweight models (YOLOv8n, YOLOv8s) are characterised more rigorously over additional random seeds, a training-data ablation, and a per-subclass analysis in the cross-validated study of Table 6. For industrial deployment with limited labelled data, the lightweight YOLOv8 variants are therefore the preferred choice, combining competitive detection quality with the lowest computational footprint; YOLOv8n in particular exhibits the most consistent behaviour across folds.

### Comparison with related work

Table 10 compares our results with representative studies on deep learning-based industrial inspection. Our approach achieves competitive detection performance with substantially fewer training samples than most prior work. While [8] required 320–640 samples to reach 94.5% *mAP* on WAAM defect detection, and [2] used over 1,000 samples for smart manufacturing anomaly detection, the proposed YOLOv8n model achieves *0.842 ± 0.050* *mAP@0.5* with just 40 samples per class, evaluated under cross-validation. [11] achieve comparable performance with even fewer samples per class but on a classification task without localisation, which is a strictly easier problem. Unlike most existing studies that report results for a single training configuration, our analysis varies the training set size and reports cross-validated performance, providing the empirical scaling guidance that has been absent from the literature. The comparison reinforces two qualitative findings. First, architecture selection has a larger impact on data efficiency than incremental data collection: the YOLOv8 family achieves strong performance with minimal data, while YOLOv5 variants require substantially more training samples. Second, hybrid real-plus-synthetic data is a viable alternative when real *Abnormal* samples are scarce, and the ablation in Section 5.3 quantifies its contribution.

**Table 10:** Comparison with related work on deep learning-based industrial inspection. Our result is reported as mean ± std under 5-fold cross-validation

Study	Application	Samples	Best Result	Synthetic + Real
[25]	Surface morphology	*∼*48	62.04% *mAP*	✗
[8]	WAAM defects	320–640	94.5% *mAP*	✗
[26]	Assembly inspection	20–160	96.7% *mAP*	✗
[7]	Construction	1k–10k	69.7% *AP*	✓
[2]	Smart manufacturing	1k–2.6k	98.9% *P*	✗
[11]	Metal defects (few-shot)	5–20/class	91.6% Acc	✗
This work	LS inspection	40/class	*0.842 ± 0.050* *mAP@0.5*	✓

## Discussion

Based on our experimental findings, we present the following suggestions for deploying deep learning-based visual inspection in industrial settings:

**Minimum training data:** For binary inspection tasks with clear visual cues in controlled environments, 40 labelled images per class (80 total) are sufficient for the lightweight YOLOv8 variants (YOLOv8n, YOLOv8s) to achieve reliable detection performance, with YOLOv8n reaching *0.842 ± 0.050* *mAP@0.5* at *T*-40 and *0.946 ± 0.047* *mAP@0.5* at *T*-50. YOLOv5 variants require approximately 100 samples per class to approach comparable performance.

**Architecture selection:** YOLOv8 is the preferred family for data-scarce industrial applications. Within YOLOv8, the nano variant (YOLOv8n) offers the best Recall and F1 across all training sizes evaluated, while YOLOv8s provides comparable accuracy with slightly higher computational cost. Both lightweight variants outperform their larger counterparts and the YOLOv5 family.

**Model capacity:** Smaller models including YOLOv8n and YOLOv8s outperform larger variants such as YOLOv8m, YOLOv8l, and YOLOv8x in data-scarce settings. Larger models may overfit and generalise poorly when training data are limited; this is consistent with the convergence behaviour observed in Section 5.2.

**Hybrid training data:** Combining real *Normal* and real misaligned samples with synthetic obstructions is essential for robust performance. Our ablation study (Section 5.3) shows that removing either source approximately halves Recall, with each source contributing complementary subclass coverage: synthetic obstructions cover $\mathcal {A}_1$ and $\mathcal {A}_2$ effectively, while real $\mathcal {A}_3$ samples are required to capture the misaligned-door condition.

**Confidence threshold:** A confidence threshold of 0.9 is appropriate for quality-critical applications. Combined with the rules described in Section 4.1, this ensures that only high-certainty predictions are accepted as *Normal*, while uncertain cases are flagged for review.

**Transfer learning:** All models were initialised from COCO pretrained weights. The strong performance with limited data is directly attributable to transfer learning. Training from scratch is not recommended when fewer than several hundred labelled images are available.

Some limitations should be considered when interpreting these results. First, the misaligned subclass ($\mathcal {A}_3$) recall of approximately 0.64 at *T*-40 is constrained by the availability of only 15 real misaligned samples per training fold, rather than by detection capacity. Increasing $\mathcal {A}_3$ training data would likely improve this subclass without altering the qualitative findings on other subclasses. Second, several configurations exhibit considerable cross-validation variance (e.g. standard deviations above 0.20 on Recall), reflecting the inherent fold-to-fold sensitivity of small training pools and the practical implications for deployment reproducibility. Third, all experiments were conducted on Load Station inspection in semiconductor manufacturing under controlled lighting and a stable camera position; generalisation to other inspection domains, lighting conditions, or camera configurations requires separate validation. Finally, this study focuses on binary classification; multi-class scenarios that distinguish among $\mathcal {A}_1$, $\mathcal {A}_2$, and $\mathcal {A}_3$ as separate output categories may require additional training data to achieve comparable per-class performance.

## Conclusion

This study investigates deep learning-based Load Station (LS) inspection for semiconductor wafer handling under realistic data scarcity. By varying training set sizes and comparing different YOLO architectures under cross-validated evaluation, we provide statistically grounded guidelines for deploying visual inspection systems with limited labelled industrial images.

### Minimum data requirements:

Cross-validated experiments show that reliable LS inspection can be achieved with as few as 40 labelled samples per class using transfer learning. YOLOv8n achieved *0.981 ± 0.014* accuracy and *0.842 ± 0.050* *mAP@0.5* at *T*-40, rising to *0.946 ± 0.047* *mAP@0.5* at *T*-50. YOLOv5 configurations required approximately *T*-100 to reach comparable accuracy. With appropriate architecture selection, fewer than 100 well-labelled samples per class are sufficient for effective model training.

### Architecture selection:

Among the evaluated architectures, the lightweight YOLOv8 variants (n, s) provide the best balance of accuracy, recall, and data efficiency. YOLOv8n delivered the strongest detection performance across training sizes, while YOLOv8s offered comparable accuracy with slightly higher computational cost. YOLOv5 variants required substantially more training data to achieve similar performance.

### Model capacity:

Contrary to the conventional expectation that larger models perform better, smaller variants (YOLOv8n, YOLOv8s) outperformed larger ones (YOLOv8m, YOLOv8l, YOLOv8x) under data-scarce conditions. The larger variants showed substantially lower performance under preliminary cross-validated evaluation, consistent with overfitting on limited training data. Lightweight architectures are therefore preferable for industrial deployment with constrained datasets.

### Hybrid data effectiveness:

The combination of real *Normal* samples with real misaligned ($\mathcal {A}_3$) and synthetically generated obstruction samples proved essential for robust inspection. The ablation study (Table 7) confirmed that removing either real or synthetic abnormal sources approximately halves Recall, with the two sources providing complementary subclass coverage. The hybrid approach addresses the practical challenge that abnormal conditions are rare in operational environments and cannot be artificially induced without disrupting production.

### Real-world viability:

The approach was validated on an operational semiconductor manufacturing dataset, distinguishing *Normal* from *Abnormal* Load Station states across three abnormal subclasses (cassette present, foreign objects on the LS surface, and LS misalignment). The cross-validated results demonstrate that deep learning-based visual inspection can be integrated into existing manufacturing workflows with minimal disruption, and that transfer learning from generic datasets adapts successfully to specialised industrial tasks.

Future research should focus on evaluating this approach across different Load Station configurations and lighting conditions to assess broader generalisation, integrating with digital twin frameworks for predictive maintenance, and investigating continual learning strategies for adaptive inspection systems. Expanding the real $\mathcal {A}_3$ sample budget would also be valuable to close the per-subclass gap identified in Section 5.4. Together, these directions move toward fully autonomous smart manufacturing.

## Data Availability

The datasets generated and analysed during the current study are not publicly available due to confidentiality agreements with the industrial partner however are available from the corresponding author on reasonable request.
